# The combination of rib osteosynthesis and simultaneous operations at patients with flail chest

**DOI:** 10.1186/1749-8090-8-S1-O72

**Published:** 2013-09-11

**Authors:** A Benyan, S Pushkin

**Affiliations:** 1 Samara Regional Clinical Hospital, Samara, Russian Federation

## Background

The main principles in management of patients with severe blunt chest trauma are repair of internal injuries and stabilization of chest. The patients with flail chest are needed in simultaneous operations on ribs and pleural cavity.

## Methods

We have observed 10 consecutive patients with flail chest at 1,5-year period. They were 7 male, 3 female. The age was 42-71 years. Four patients also had multiple injuries and underwent operations on other organs. The terms before thoracic operation were 24 – 72 hours. Number of fractured ribs was from 4 to 10. The fractures of right ribs were at 3 patients, of left ribs – at 2 patient, bilateral rib fractures had 5 patients. Hemopneumothorax was observed at 5 patients, lung laceration – at 2 patients, fracture of sternum – at 2 patient, fracture of clavicle – at 1 patient, rupture of left cupula of diaphragm – at 1 patient. All patients had different degree of respiratory insufficiency, 3 of them had ARDS.

## Results

To all of these patients we have performed simultaneous operations on ribs and organs of pleural cavity. For rib osteosynthesis we used Matrix Rib technologies. The number of synthesized ribs was from 2 to 5. At patients with bilateral fractures we performed appropriate bilateral osteosynthesis. In accordance with presenting injuries we have performed 5 thoracoscopic sanation of hemothorax, 1 thoracoscopic suturing of diaphragm, 2 thoracotomy and suturing of lung, 1 osteosynthesis of sternum, 1 osteosynthesis of clavicle, 1 tracheostomy. There were no lethal outcomes. The average of ventilation days was 2,2. We didn`t observe any significant respiratory or wound complications. The postoperative period was 7-21 days. All patients were discharged from hospital in satisfactory condition.

## Conclusions

Performance of simultaneous rib osteosynthesis and operations on the injured organs of pleural cavity is required at patients with flail chest.

